# Computational prediction of disease microRNAs in domestic animals

**DOI:** 10.1186/1756-0500-7-403

**Published:** 2014-06-27

**Authors:** Teresia Buza, Mark Arick, Hui Wang, Daniel G Peterson

**Affiliations:** 1 Department of Basic Sciences, College of Veterinary Medicine, Mississippi State University, P. O. Box 6100, Mississippi State 39762, USA; 2 Institute for Genomics, Biocomputing & Biotechnology, Mississippi State University, P. O. Box 9627, Mississippi State 39762, USA

**Keywords:** Disease microRNAs, Target, Domestic animals, Homology, Orthology, Phylogenetic analysis

## Abstract

**Background:**

The most important means of identifying diseases before symptoms appear is through the discovery of disease-associated biomarkers. Recently, microRNAs (miRNAs) have become highly useful biomarkers of infectious, genetic and metabolic diseases in human but they have not been well studied in domestic animals. It is probable that many of the animal homologs of human disease-associated miRNAs may be involved in domestic animal diseases. Here we describe a computational biology study in which human disease miRNAs were utilized to predict orthologous miRNAs in cow, chicken, pig, horse, and dog.

**Results:**

We identified 287 human disease-associated miRNAs which had at least one 100% identical animal homolog. The 287 miRNAs were associated with 359 human diseases referenced in 2,863 Pubmed articles. Multiple sequence analysis indicated that over 60% of known horse mature miRNAs found perfect matches in human disease-associated miRNAs, followed by dog (50%). As expected, chicken had the least number of perfect matches (5%). Phylogenetic analysis of miRNA precursors indicated that 85% of human disease pre-miRNAs were highly conserved in animals, showing less than 5% nucleotide substitution rates over evolutionary time. As an example we demonstrated conservation of human *hsa-miR-143-3p* which is associated with *type 2 diabetes* and targets AKT1 gene which is highly conserved in pig, horse and dog. Functional analysis of AKT1 gene using Gene Ontology (GO) showed that it is involved in glucose homeostasis, positive regulation of glucose import, positive regulation of glycogen biosynthetic process, glucose transport and response to food.

**Conclusions:**

This data provides the animal and veterinary research community with a resource to assist in generating hypothesis-driven research for discovering animal disease-related miRNA from their datasets and expedite development of prophylactic and disease-treatment strategies and also influence research efforts to identify novel disease models in large animals. Integrated data is available for download at http://agbase.hpc.msstate.edu/cgi-bin/animal_mirna.cgi.

## Background

MicroRNAs (miRNAs) are naturally occurring single-stranded small RNA molecules that play important roles in post-transcriptional regulation of gene expression [1]. Studies have shown that miRNAs exert their regulatory role by partially binding their target (complementary) mRNAs at 3′ UTRs (untranslated regions) [2-5]. Phylogenetic studies indicate that animal miRNAs are highly conserved [6,7]. Until recently, miRNAs were thought to be of little or no cellular significance [8-10]. The first miRNA shown to have a regulatory function was lin-4 from *Caenorhabditis elegans*[11,12]. Lin-4 acted as a silencer of genes that regulate developmental timing, but it was considered a unique evolutionary adaptation as lin-4 homologs were not found in other species. The discovery of the regulatory miRNA let-7 in *C. elegans* in 2000 [10], with homologs in other species including humans, caused researchers to reconsider the idea that miRNAs may have a more widespread function within cells. We now know that many miRNAs play central roles in post-transcriptional gene regulation. Additionally, expression of specific miRNAs has been linked to various diseases [13-16]. Considerable research has been devoted to understanding regulatory roles of miRNAs in human diseases [17-26], and miRNAs are important biomarkers of several disease processes [27-32] including many cancers [33-43] and cardiovascular [18,44-53], inflammation [54-57], and gastrointestinal diseases [58-60].

**Figure 1:**
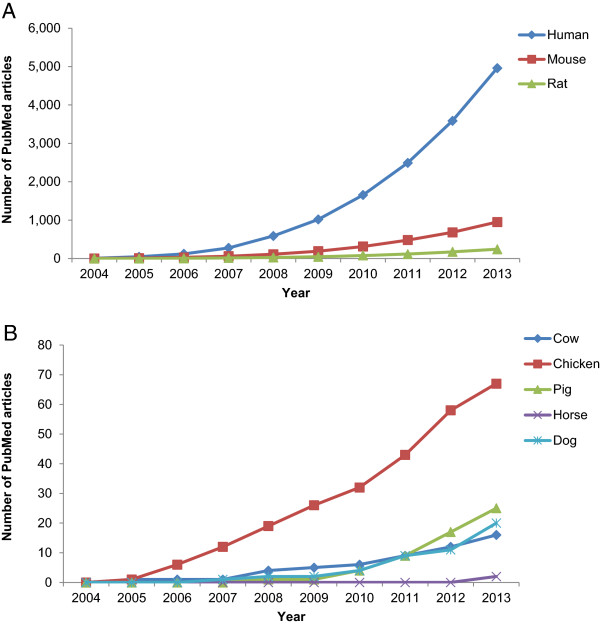
**Publication statistics of disease associated miRNAs as of 12/31/2013.** Searches from NCBI-Pubmed titles or abstracts were conditioned to retrieve publications from the last ten years. The searches contained species names, miRNA abbreviations, disease(s) and duration (year). For example searching Pubmeds for dog disease associated miRNAs in the past 10 years were acquired using the following query statement: (dog OR canine OR (Canis familiaris) AND (microRNA OR microRNAs OR miRNA OR mirRNAs OR mir OR miRs) AND (cancer OR cancers OR disease OR diseases OR disorder OR disorders) AND ((“2004/01/01”[PDat] : “2013/12/31”[PDat])). Note that **(A)** and **(B)** are presented in different y-axis scale due to large difference in number of Pubmed articles.

While databases for human disease-associated miRNAs are publicly available [61-70], there is no any integrated resource for disease-associated miRNAs in domestic animals. An integrated resource of animal disease-related miRNA data would provide the animal and veterinary research community with an invaluable resource for searching disease related miRNA subsets from their experimental data. Pubmed articles stand solely as the major reliable source of information for disease miRNA data. However, there are very few Pubmed articles currently (as of 12/31/2014) documenting disease-associated miRNAs in domestic animals compared to human, mouse or rat (Figure 1). Identification of miRNA/disease associations in domestic animals is critical for understanding miRNA involvement in the pathophysiology of these organisms.

The main objective of our study was to identify animal miRNA homologs of published human disease-associated miRNAs in cow, chicken, pig, horse and dog using phylogenetic techniques. Using the current available human and animal miRNA resources, we identified potential disease-related miRNAs in domestic animals based on integrated computational and manual approaches including assessing the sequence similarities and evolutionary relationships between human disease miRNAs and their animal orthologs. These predictions will serve as a resource to facilitate hypothesis-driven research in domestic animals, which upon verification in animals could suggest animal models for human diseases and strategies for developing therapeutic measures.

## Methods

### Human and domestic animal mature miRNA sizes

We compared the sizes of all known mature human miRNAs with the sizes of all known cow, chicken, pig, horse, and dog to establish their length diversity.

### Extraction and verification of disease-associated human miRNAs

Briefly, we surveyed publicly available databases that link human miRNAs with diseases [65,67,71,72] and selected the most up-to-date and comprehensive human disease miRNA database, i.e., the Human miRNA Disease Database (HMDD) version 2.0 [58], as a baseline for searching animal homologs. We then filled a request form available at http://202.38.126.151/hmdd/html/tools/hmdd_req.html to request all human disease-associated miRNA data from Dr. Qinghua Cui of department of Biomedical Informatics, Peking University Health Science Center. From this data we retrieved the miRNA IDs (identifiers) that were named according to miRBase [73] nomenclature standards, the disease(s) associated with each miRNA and the Pubmed articles from which the HMDD data was extracted. We then manually reviewed the Pubmed titles and abstracts to verify association of the miRNAs with human diseases.

### Prediction of animal homologs of human disease-associated miRNAs

We used the IDs of human disease miRNAs (from HMDD) to extract corresponding mature (i.e., processed) miRNA nucleotide sequences from miRBase version 20 [73]. We also downloaded all sequences of mature and precursor (pre) miRNAs for cow, chicken, pig, horse and dog from miRBase version 20 and then used a Perl script to identify cow, chicken, pig, horse, and dog mature miRNA sequences that were 100% identical to sequences of human disease-associated miRNAs. The outputs of the Perl script were deemed “human disease miRNAs with animal counterparts” (HDMACs).

### Phylogenetic analysis of HDMAC precursor sequences

The precursor miRNA sequences (pre-miRNAs) of HDMACs were compared using a multiple alignment and phylogenetic approach to detect conservation profiles and rapid sequence divergence in human and domestic animals. Briefly, we used Clustal Omega [74] tool for multiple alignment of pre-miRNA sequences and Clustalw2-Phylogeny tool [75] to generate the Neighbor-Joining (NJ) phylogenetic trees to determine nucleotide substitutions that have occurred over evolutionary time. Briefly, NJ method compares each sequence with each other, calculates distance matrices, then combines the least distant pair of sequences and construct phylogenetic tree. We displayed distances (divergence proportions) calculated from all pairs of sequences in the multiple alignments to facilitate evolutionary interpretation of phylograms. Divergence proportions less or equal to 5% (≤0.05) were considered to be highly conserved.

### Annotation of human disease miRNA targets and their animal orthologs

We manually annotated the genes targeted by the human disease-associate miRNAs from the associated Pubmed articles and predict their animal orthologs using the Ensembl Biomart [76] tool. All human targets and their animal orthologs with one-to-one matches and reciprocal% identity >70 were integrated in the animal disease miRNA resource.

### Integration of disease information with human-animal miRNA homologs

We integrated the information gathered from this study to form a computationally predicted animal disease miRNA resource which contained miRNA information including standardized miRBase identifiers of mature miRNAs, pre-miRNAs, and miRNA families linked to the associated human disease. Whenever applicable we used OMIM (Online Mendelian Inheritance in Man) [77], OMIA (Online Mendelian Inheritance in Animal) [78] and Disease Ontology (DO) [79] standardized names for disease phenotypes and BRENDA (BRaunschweig ENzyme DAtabase) Tissue Ontology (BTO) [80,81] terms to standardize names of source tissues or cell types. Pubmed IDs were used as central literature references. Additional information regarding the genomic location(s) of miRNAs, type of experiment, and publication date was also integrated.

## Results

### Lengths of animal and human mature miRNAs

**Figure 2:**
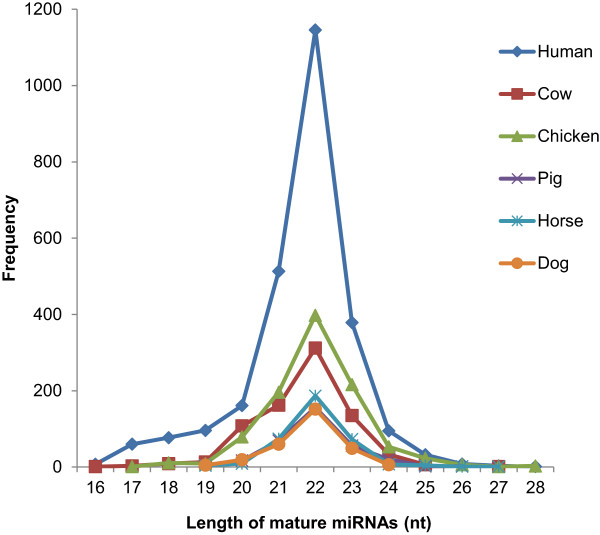
**An overall distribution of sequence lengths of mature miRNAs.** The length corresponds to the number of nucleotides in a miRNA sequence.

Generally, the distribution of mature miRNA lengths in all species followed same trend, with 22-nt sequences dominating in each species (Figure 2).

### Dataset of human disease-associated miRNAs

**Figure 3:**
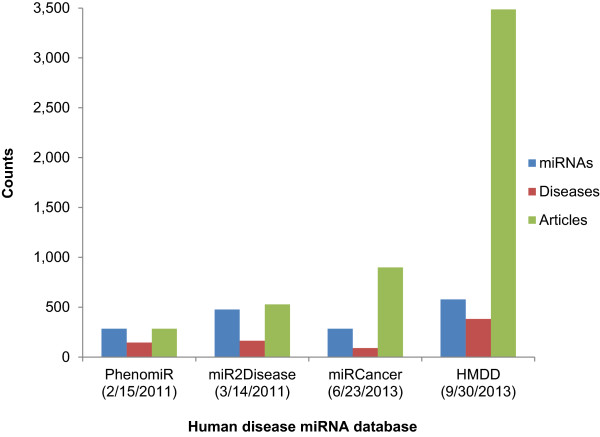
**Statistics of human disease miRNA databases as of 12/31/2013.** The dates in the brackets indicate the last time the database was updated.

After surveying various human disease-related miRNA databases, we found that the miRNAs in the Human miRNA Disease Database (HMDD) version 2.0 [71] (updated on 09/30/2013) were best suited for use as a training set (Figure 3). This database contained 578 published human miRNAs associated with 383 diseases referenced in 3,486 Pubmed articles. The human disease miRNA referenced in HMDD included 70% of the total 4,961 human disease miRNA articles we identified in Pubmed (Figure 1).

**Figure 4:**
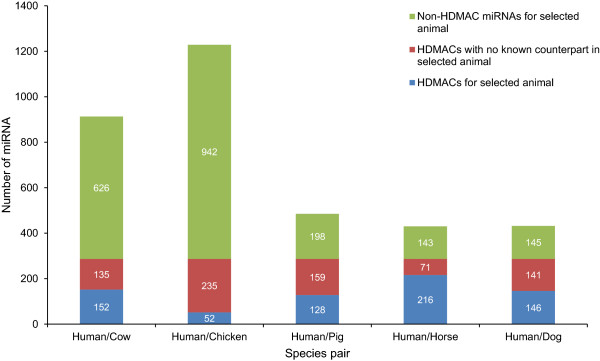
**Mature human disease miRNA with animal counterparts (HDMACs) in key domestic animals.** For a particular human-animal pair, blue bar indicates HDMACs in other animals but not in the selected animal, red bars indicates HDMACs in the select animal and blue & red bars together represent 287 HDMACs. Green sub-bars show non-HDMACs.

We identified 694 domestic animal mature miRNA sequences that showed 100% sequence identity with the 287 human disease miRNAs (Figure 4, Additional file 1). Over 60% of total horse mature miRNAs (216) showed perfect matches to human disease-associated miRNAs, followed by dog (50%). As expected, chicken had the least number of perfect matches (5%), most likely a result of the relative evolutionary distance between birds and mammals. We found that 14 human disease miRNAs were conserved in all animals, chicken included, while 41 were conserved only between the mammalian representatives (Table 1).

**Table 1:** Human disease miRNA with animal counterparts

**Human_homolog**	**MicroRNA family**	**Cow (bta)**	**Chicken (gga)**	**Pig (ssc)**	**Horse (eca)**	**Dog (cfa)**	**Total animal species**
hsa-miR-128-3p	mir-128	+	+	+	+	+	5
hsa-miR-130a-3p	mir-130	+	+	+	+	+	5
hsa-miR-135a-5p	mir-135	+	+	+	+	+	5
hsa-miR-148a-3p	mir-148	+	+	+	+	+	5
hsa-miR-184	mir-184	+	+	+	+	+	5
hsa-miR-19a-3p	mir-19	+	+	+	+	+	5
hsa-miR-193a-5p	mir-193	+	+	+	+	+	5
hsa-miR-204-5p	mir-204	+	+	+	+	+	5
hsa-miR-205-5p	mir-205	+	+	+	+	+	5
hsa-miR-27b-3p	mir-27	+	+	+	+	+	5
hsa-miR-29b-3p	mir-29	+	+	+	+	+	5
hsa-miR-30b-5p	mir-30	+	+	+	+	+	5
hsa-miR-365b-3p	mir-365	+	+	+	+	+	5
hsa-miR-490-3p	mir-490	+	+	+	+	+	5
hsa-miR-19b-3p	mir-19	+	+	+	+	-	4
hsa-miR-193a-3p	mir-193	+	+	+	+	-	4
hsa-miR-24-3p	mir-24	+	+	+	+	-	4
hsa-miR-206	mir-1	+	+	-	+	+	4
hsa-miR-20a-5p	mir-17	+	+	-	+	+	4
hsa-miR-190a-5p	mir-190	+	+	-	+	+	4
hsa-miR-194-5p	mir-194	+	+	-	+	+	4
hsa-miR-216b-5p	mir-216	+	+	-	+	+	4
hsa-miR-383-5p	mir-383	+	+	-	+	+	4
hsa-miR-98-5p	let-7	+	-	+	+	+	4
hsa-miR-99b-5p	mir-10	+	-	+	+	+	4
hsa-miR-130b-3p	mir-130	+	-	+	+	+	4
hsa-miR-148b-3p	mir-148	+	-	+	+	+	4
hsa-miR-149-5p	mir-149	+	-	+	+	+	4
hsa-miR-187-3p	mir-187	+	-	+	+	+	4
hsa-miR-532-5p	mir-188	+	-	+	+	+	4
hsa-miR-196a-5p	mir-196	+	-	+	+	+	4
hsa-miR-208b-3p	mir-208	+	-	+	+	+	4
hsa-miR-92a-3p	mir-25	+	-	+	+	+	4
hsa-miR-92b-3p	mir-25	+	-	+	+	+	4
hsa-miR-26a-5p	mir-26	+	-	+	+	+	4
hsa-miR-151a-5p	mir-28	+	-	+	+	+	4
hsa-miR-29c-3p	mir-29	+	-	+	+	+	4
hsa-miR-324-5p	mir-324	+	-	+	+	+	4
hsa-miR-328-3p	mir-328	+	-	+	+	+	4
hsa-miR-331-3p	mir-331	+	-	+	+	+	4
hsa-miR-34a-5p	mir-34	+	-	+	+	+	4
hsa-miR-361-5p	mir-361	+	-	+	+	+	4
hsa-miR-376a-3p	mir-368	+	-	+	+	+	4
hsa-miR-370-3p	mir-370	+	-	+	+	+	4
hsa-miR-374b-5p	mir-374	+	-	+	+	+	4
hsa-miR-423-5p	mir-423	+	-	+	+	+	4
hsa-miR-499a-5p	mir-499	+	-	+	+	+	4
hsa-miR-628-5p	mir-628	+	-	+	+	+	4
hsa-miR-708-5p	mir-708	+	-	+	+	+	4
hsa-miR-885-5p	mir-885	+	-	+	+	+	4
hsa-miR-146a-5p	mir-146	-	+	+	+	+	4
hsa-miR-21-5p	mir-21	-	+	+	+	+	4
hsa-miR-218-5p	mir-218	-	+	+	+	+	4
hsa-miR-22-3p	mir-22	-	+	+	+	+	4
hsa-miR-34c-5p	mir-34	-	+	+	+	+	4
hsa-miR-9-5p	mir-9	-	+	+	+	+	4

A complete table of all matches is provided in the Additional file 1.

### Conserved human disease pre-miRNAs in animals

**Figure 5:**
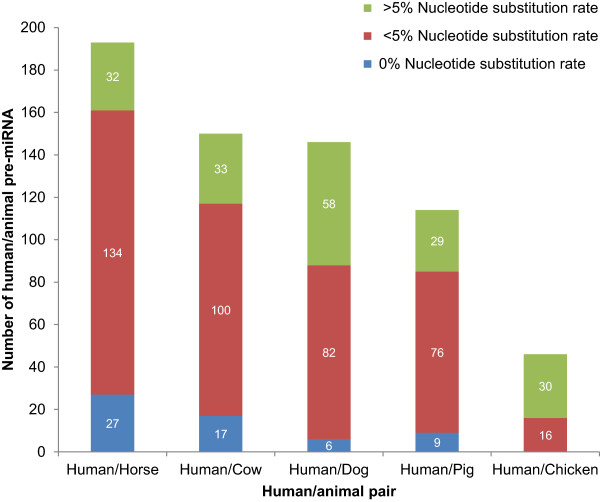
**Nucleotide substitution rate of pre-miRNAs of HDMACs in key domestic animals.** For a particular human-animal pair, blue indicates pre-miRNA sequences with zero nucleotide substitution rate; red bars indicates pre-miRNA sequences with <5% nucleotide substitution rate and green bars are pre-miRNA sequences with >5% nucleotide substitution rate.

**Figure 6:**
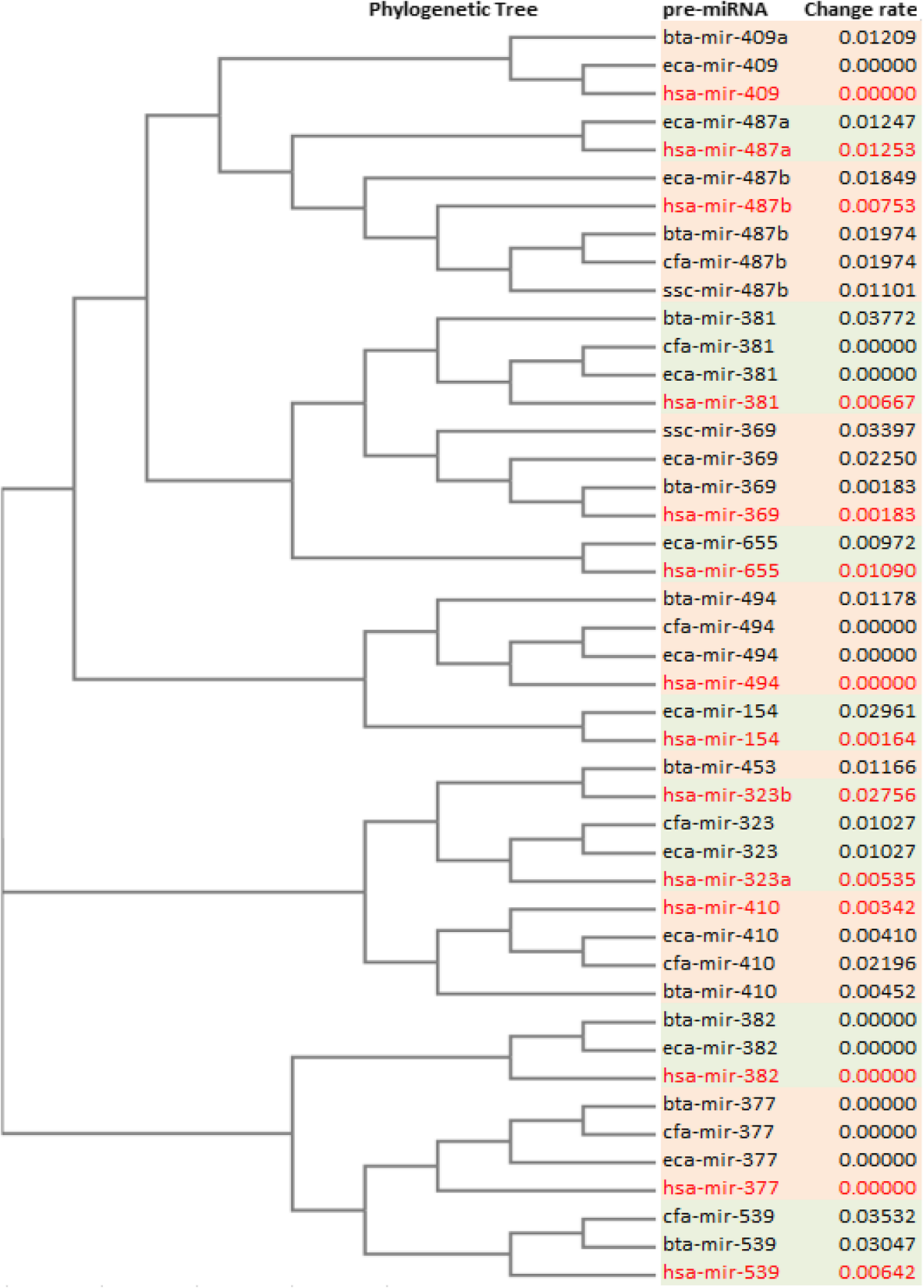
**Example of phylogenetic analysis of HDMACs pre-miRNAs in the mir-154 family.** The branch labels are pre-miRNA IDs prefixed by abbreviation of species scientific name; *hsa* (*Homo sapiens* - human), *bta* (*Bos Taurus* - cow), *gga* (*gallus gallus* - chicken), *ssc* (*Sus scrofa* - pig), *eca* (*Equine canibus* - horse), and *cfa* (*Canis familiaris* - dog); followed by numbers that indicate the proportion of evolutionary distance in terms of nucleotide substitutions per site per unit time, which indicates the changes in sequences when they evolved from a common ancestral sequence. The alternating background colors (light pink, light green) facilitate visualization and comparison of miRNA species within the cluster.

In addition to producing multiple alignments of mature miRNAs, we established additional evolutionary relationship between pre-miRNAs of HDMACs, which included 265 and 649 human and animal pre-miRNA sequences, respectively. About 85% of human disease pre-miRNAs were found to be highly conserved in animals, showing less than 5% nucleotide substitution rates over evolutionary time (Figure 5; Additional file 2). We demonstrate phylogenetic analysis of pre-miRNA sequences of HDMACs that are clustered in the mir-154 family (Figure 6). In this family all pre-miRNAs had nucleotide substitution rate of <4%, among which, pre-miRNA sequences of eight animals; 4 in horse (eca-mir 409, 494, 377 & 382), 2 in dog (cfa-mir-494 & 377) and 2 in cow (bta-mir-377 & 382) had 100% percent identity with 4 of human disease pre-miRNAs sequences.

### Animal orthologs of human disease miRNA targets

**Figure 7:**
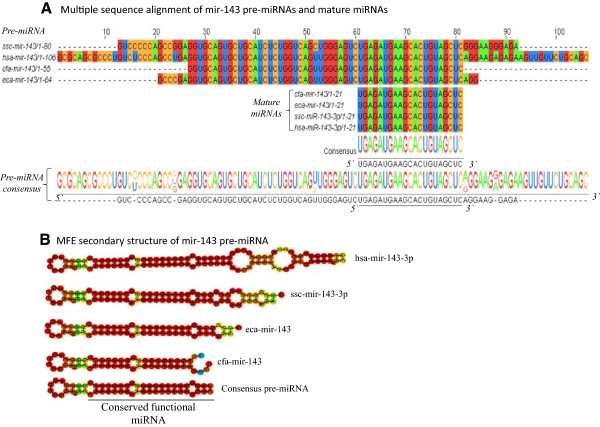
**Comparative structural analysis of human *hsa-miR-143 *and its animal orthologs.** The multiple sequence alignment **(A)** shows the location of the mature miRNA on the 3′ arm of the pre-miRNA. The minimum free energy (MFE) secondary structure **(B)** of human *mir-143* is compared to that of the animal orthologs and a normalized pre-miRNA consensus sequence. The species are abbreviated by their scientific names; hsa (Homo sapiens –human), *ssc* (*Sus scrofa* - pig), *eca* (*Equine canibus* - horse), and *cfa* (*Canis familiaris* - dog).

Genes targeted by the human disease-associated miRNAs were utilized to predict their animal orthologs using the Ensembl Biomart [76] tool. Currently, over 45 targets conserved across human and domestic animals are included in the integrated resource available through AgBase [82,83] at http://agbase.hpc.msstate.edu/cgi-bin/animal_mirna.cgi. Example of conserved disease miRNA targets linked to type 2 diabetes is included in this article (Figure 7, Table 2) and more targets are shown as Additional file 3.

**Table 2:** **Comparative functional analysis of confirmed target of human *hsa-mir-143-3p *** **and predicted target of animal orthologs**

**Species**	**Functional mature miRNA**	**Target gene symbol**	**Target gene length**	**% identity human-animal AKT1 (full-length alignment)**	AKT1 predicted biological processes (UniProtKB GO)*	**mir-143 target inhibition**
Human	hsa-miR-143-3p	AKT1 (Confirmed)	480 aa	100	Glucose homeostasis, positive regulation of glucose import, positive regulation of glycogen biosynthetic process, response to food, glucose transport.	Cleavage
Pig	ssc-miR-143-3p	AKT1 (Predicted)	480 aa	100	Glucose homeostasis, positive regulation of glucose import, positive regulation of glycogen biosynthetic process, response to food, glucose transport.	Cleavage
Horse	eca-miR-143	AKT1 (Predicted)	479 aa	98	Glucose homeostasis, positive regulation of glucose import, positive regulation of glycogen biosynthetic process, response to food, glucose transport	Cleavage
Dog	cfa-miR-143	AKT1 (Predicted)	479 aa	97	Glucose homeostasis, positive regulation of glucose import, positive regulation of glycogen biosynthetic process, response to food, glucose transport	Cleavage

Over-expression of *hsa*-*miR-143* inhibits insulin-stimulated AKT activation and impairs glucose metabolism [83].

*Showing only the biological process GO terms with direct relevance to type 2 diabetes.

### Data integration

In order to provide a unified view of data generated from this study we integrated all information to form a core resource of domestic animal disease-related miRNAs. The integrated data links all 694 animal mature miRNAs with 287 human miRNAs which are associated with 359 human diseases referenced in 2,863 Pubmed articles. This information is classified into five main categories including information for miRNA, associated disease, reference, genomic location and target (Table 3). The integrated resource is the main reference and preliminary data towards our efforts to develop an advanced farm and domestic animal disease-associated miRNA resource. The preliminary integrated resource is available at http://agbase.hpc.msstate.edu/cgi-bin/animal_mirna.cgi.

**Table 3:** Summary of integrated information in the animal disease-miRNA resource

**Information**	**Specific information**
MicroRNA	1. Mature miRNA ID
	2. Mature miRNA AC
	3. Mature RNA sequence
	4. Pre-miRNA/Hairpin ID
	5. Pre-miRNA/Hairpin AC
	6. Pre-miRNA/Hairpin sequence
	7. Family ID
	8. Family AC
Location	9. Chromosome number
	10. Genomic coordinates (start & end)
	11. Strand (positive or negative)
Reference	12. Pubmed ID
	13. Pubmed Central ID (indicates free articles)
	14. Pubmed title
	15. Pubmed publication date
Disease	16. Disease phenotype (reported in Pubmed article)
	17. Relevance in animal (for non-domestic animal diseases)
	18. OMIM/OMIA disease phenotype name
	19. OMIM/OMIA disease phenotype ID
	20. Disease Ontology (DO)
	21. Tissue/Cell type (source of samples used)
	22. Brenda Tissue Ontology (BTO) name
	23. BTO ID
	24. Method used to associate a disease with a miRNA
Target	25. Gene targeted by the disease miRNA
	26. Animal ortholog of targeted gene
	27. Reciprocal% identity of target:ortholog
	28. Orthology_confidence[0 low,1 high]

The integrated data is availableat http://agbase.hpc.msstate.edu/cgi-bin/animal_mirna.cgi.

### Application of the integrated resource

We have demonstrated how experimentally confirmed *diabetes type 2-associated miRNA hsa-mir-143-3p* can be used to identify related miRNAs in animals (Figure 7, Table 2) thus, providing a more focused hypothesis-driven investigations in animals. We found that the *hsa-miR-143* which is located on the right arm (3′) of its pre-miRNA is highly conserved in pig, horse and dog. The *hsa-miR-143-3p* target gene is AKT1 [84] which has 97- 100% sequence identity with AKT1 found in pig, horse and dog. The biological processes annotated to AKT1 using Gene ontology (GO) [85,86] indicate that this gene is involved in similar processes in all species including, glucose homeostasis, positive regulation of glucose import, positive regulation of glycogen biosynthetic process, response to food and glucose transport (Table 2).

## Discussion

One means of identifying diseases before symptoms appear is through the discovery and utilization of disease-associated molecular biomarkers. Many biomarker techniques that have been widely applied in human and model organism studies have not been adequately implemented in the study of domestic animal diseases. It is now clear that miRNA play major regulatory role in various disease processes but financial investment is more committed to investigate miRNA involvement in human disease more than any other species (Figure 1). There are several miRNAs currently classified as biomarkers for human cancers [27,42,87-93], cardiovascular [45,46,94,95], and inflammation [54-57] diseases. Although not experimentally found to be associated with miRNAs, some of these human diseases are also found in domestic animals [96-99]. Advances in knowledge about human disease-associated miRNAs warrant investigation of similar diseases in related species.

In this study we used homology approach to generate a resource that integrates animal miRNA data with human disease-associated miRNAs. As demonstrated using *hsa-miR-143-3p* which has been associated with diabetes type 2 [84] (Figure 7 and Table 2) it is logical that similar miRNAs perform comparable functions across related species, and therefore diseases correlated with miRNAs in one species may be correlated with homologous miRNA expression and disease in related species. The example of diabetes type 2-associated miRNA hsa-miR-143-3p gave a highlight on how to link disease-associated elements across species and develop hypothesis-driven investigation in animals. Integrating all data enabled us to identify some human disease miRNAs that are found in more than one animal species (Additional file 1), which indicates the likelihood of also sharing common diseases. Having miRNAs targeting similar genes across species provide clue of functional orthology. As indicated in this study one miRNA can be associated with multiple diseases. For example, hsa-miR-21-5p has been documented in nearly 400 Pubmed articles and is associated with 124 human disease phenotypes and has homologs in four animals including chicken.

However, not all human miRNA-related diseases may be relevant to all animals. Manual curation effort to continue building and updating the animal disease miRNA resource developed in this study is our long term process. The relevance of each human miRNA disease linked to each animal and targeted gene will continue to be established and integrated in the resource. Validation of the human disease miRNAs in the animal context will likely leverage the findings in human at the same time improve our understanding of their involvement in the pathogenesis, diagnosis, and prognosis of various animal diseases.

## Conclusions

In this study we have shown that some human disease-associated miRNAs are well conserved across domestic animals. Also, human genes targeted by disease-associated miRNAs are highly conserved in animals. Conservation of both miRNAs and their target genes across human and domestic animals provides the likelihood of having functional orthology relationship which may also lead to similar diseases. Findings from this study are a step forward towards building an advanced animal disease miRNA resource, identifying miRNA-related diseases in animals and utilization of miRNA disease biomarkers in animal and veterinary research. In the long-term, validating these human disease miRNAs in domestic animals could identify new large animal models of diseases and most likely biomarkers to expedite development of therapeutic measures for human and animal diseases.

### Availability of supporting data

The data supporting the results of this article is included within the article and its additional files. The integrated animal disease miRNA resource is freely available for download via AgBase at http://agbase.hpc.msstate.edu/cgi-bin/animal_mirna.cgi.

### Ethical requirements

Our study used human and animal data from publicly available databases and did not require ethics approval from the Institutional Review Board for the Protection of Human Subjects in Research (IRB) or the Institutional Animal Care and Use Committee (IACUC).

## Abbreviations

BRENDA: BRaunschweig ENzyme Database; BTO: BRENDA Tissue ontology; DO: Disease ontology; GO: Gene ontology; HDMACs: Human disease miRNAs with animal counterparts; HMDD: Human miRNA disease database; IGBB: Institute for genomics, biocomputing and biotechnology; MFE: Minimum free energy; miRNAs: MicroRNAs; NJ: Neighbor-joining; OMIA: Online mendelian inheritance in animal; OMIM: Online mendelian inheritance in man; pre-miRNAs: Precursor miRNAs; UTRs: Un-translated regions.

## Competing interest

The authors declare that they have no competing interests.

## Authors’ contributions

TB generated data for this manuscript and performed data analysis and interpretation, MA provided computational data integration assistance, HW generated standardized disease phenotype names for the integrated resource, TB and DGP wrote the manuscript. All authors read, critiqued, edited and approved the final manuscript.

## Supplementary Material

Additional file 1 **Distribution of human disease microRNAs across animal counterparts.** Multiple human diseases and locations are separated by a pipe (column 3). The (+) or (−) in columns 2–6 indicates presence or absence of homologous human disease miRNA (column 1) in the selected animal species. Click here for file

Additional file 2 **Phylogenetic distances of pre-miRNAs of HDMACs.** The data in column 1 & 3 are the pre-miRNA ID for animals and human, respectively prefixed with abbreviation of species scientific name i.e. *bta* (*Bos Taurus* - cow), *gga* (*gallus gallus* - chicken), *ssc* (*Sus scrofa* - pig), *eca* (*Equine canibus* - horse), *cfa* (*Canis familiaris* - dog), and *hsa* (*Homo sapiens* - human). Column 2 & 4 show the phylogenetic distances calculated based on nucleotide substitution rate within similar clusters. Click here for file

Additional file 3 **Sample of disease miRNAs targets and their animal orthologs.** Experimentally verified genes targeted by the disease miRNAs are manually curated from Pubmed articles and their % identity with an animal ortholog is generated via Ensembl Biomart [76]. Click here for file
